# From prenatal anxiety to parenting stress: a longitudinal study

**DOI:** 10.1007/s00737-017-0746-5

**Published:** 2017-06-21

**Authors:** A.C. Huizink, B. Menting, M.H.M. De Moor, M. L. Verhage, F.C. Kunseler, C. Schuengel, M. Oosterman

**Affiliations:** 1 Department of Clinical Developmental Psychology, Vrije Universiteit Amsterdam, Van der Boechorststraat 1, 1081 BT, Amsterdam, The Netherlands; 2 Section of Clinical Child and Family Studies, Department of Educational and Family Studies, Vrije Universiteit Amsterdam, Amsterdam, The Netherlands; 3 EMGO+ Institute for Health and Care Research, VU Medical Center, Amsterdam, The Netherlands; 4 Netherlands Institute for the Study of Crime and Law Enforcement (NSCR), Amsterdam, The Netherlands

**Keywords:** Pregnancy anxiety, Prenatal, Depression, Parenting stress

## Abstract

The objective of this study was to explore how maternal mood during pregnancy, i.e., general anxiety, pregnancy-specific anxiety, and depression predicted parenting stress 3 months after giving birth, thereby shaping the child’s early postnatal environmental circumstances. To this end, data were used from 1073 women participating in the Dutch longitudinal cohort Generations^2^, which studies first-time pregnant mothers during pregnancy and across the transition to parenthood. Women filled out the State Trait Anxiety Inventory (STAI), Pregnancy-Related Anxiety Questionnaire-revised (PRAQ-R), and Beck Depression Index (BDI) three times during pregnancy: at 12, 22, and 32 weeks gestational age. Three months postpartum, a parenting stress questionnaire was filled out yielding seven different parenting constructs. Latent scores were computed for each of the repeatedly measured maternal mood variables with Mplus and parenting stress constructs were simultaneously regressed on these latent scores. Results showed that trait anxiety and pregnancy-specific anxiety were uniquely related to almost all parenting stress constructs, taking depression into account. Early prevention and intervention to reduce maternal anxiety in pregnancy could hold the key for a more advantageous trajectory of early postnatal parenting.

## Introduction

Pregnancy and becoming a parent are two closely intertwined major life transitions, in which women and their partners experience major changes in their family and social roles, and in their own relationship (Grant et al. 2012). Adapting to these transitions can be expected to be accompanied by stress and anxiety (Lederman 1990; Oates 1989), alongside positive emotions as well. Indeed, particularly first-time pregnancy and parenthood are acknowledged as a period of increased emotional vulnerability (e.g., Austin et al. 2010; Morse et al. 2000). For new mothers, feelings of anxiety and depression during pregnancy are fairly common (Barnett and Parker 1986; Dayan et al. 2006), although for most women, these emotions are transient and diminish over time (e.g., Don et al. 2014; Huizink et al. 2014).

Nonetheless, for some women, feelings of anxiety and depression may persist throughout pregnancy and may set into motion biological, cognitive, and behavioral responses that may persist through early parenthood (Don et al. 2014). High levels of maternal anxiety and depression during pregnancy may have detrimental effects on both the mother and her child (e.g., Glover 2014; Dunkel Schetter and Tanner 2012) as these mood symptoms have been linked to preterm birth (e.g., Ding et al. 2014; Kramer et al. 2009), difficult infant temperament (Gutteling et al. 2005; Huizink et al. 2002), and longer-term child behavioral and emotional problems (e.g., O’Donnell et al. 2014; van den Bergh et al. 2005).

Despite the number of studies on this topic (for a review, see Van den Bergh et al. 2005), we still lack a deeper understanding of how prenatal exposure to maternal anxiety or depression can actually result in childhood behavioral problems after birth. Most studies to date have focused on stress physiological mechanisms that may underlie the association between high levels of prenatal maternal anxiety or depression and offspring outcomes. An abundant animal literature exists that describes how particularly exposure to maternal stress hormones (e.g., cortisol) of the hypothalamic-pituitary-adrenocortical (HPA) axis can influence fetal brain development and hence, offspring behavior (e.g., Meaney et al. 1996; Weinstock 2005). Some human studies also examined alterations in offspring HPA-axis regulation as a result of prenatal exposure to stress (Oberlander et al. 2008; de Weerth et al. 2013) as intermediate mechanism underlying adverse child behavior.

What has been neglected mostly, however, is the influence that the early postnatal environment may have on offspring behavior. More specifically, there is little research available to study how prenatal maternal mood may lead to parenting stress, which in turn, could also affect child behavior. As a first step, we set out to study how maternal mood during pregnancy may also be related to parenting stress, thereby shaping the child’s early postnatal environmental circumstances. Parenting stress has been generally defined as a perceived discrepancy between the demands of parenting and the available resources to meet those demands (Abidin 1995). The child, the parent, and child-parent interaction may all contain factors contributing to experienced parenting stress where the parent feels overwhelmed by the demands of parenthood (Abidin 1995).

While several studies (e.g., Bergman et al. 2008) have taken parenting factors into account as confounders, when looking at the predictive effect of prenatal maternal mood on child behavior, very little is known about how prenatal maternal mood may actually affect aspects of parenting, and particularly parenting stress. It can be expected that mothers who experienced high levels of stress or anxiety during pregnancy are also more susceptible to parenting stress after their baby is born. A previous study of our group showed that particularly anxiety during pregnancy was related to lower (expected) parenting self-efficacy in pregnancy as well, suggesting that anxiety during pregnancy hampers an optimal preparation for parenting (Wernand et al. 2014). These findings are in line with the observation that mothers with psychiatric problems in the postpartum period have a reduced ability to mobilize important psychological resources to cope with parenthood, and perceive parenting as more negative, leading to higher levels of parenting stress (Webster-Stratton 1998; Whiffen and Gotlib 1989). The same may apply, at least partly, for women with high levels of prenatal anxiety or depression, but little evidence is available. A relatively small clinical study of 52 selective depressed pregnant women (of whom the majority used antidepressants) in comparison to 42 non-depressed women showed that both depression and anxiety in third trimester of pregnancy were related to higher overall levels of parenting stress at 3 and 6 months postpartum (Misri et al. 2010).

We aim to extend the latter study by including a much larger sample of pregnant women and focus on different aspects of parenting stress as outcome of repeatedly measured different aspects of prenatal mood symptoms, including pregnancy anxiety, general anxiety, and depression. It may be expected that women with high levels of pregnancy-specific anxiety—including fear about giving childbirth, worries about their bodily changes due to pregnancy, and about the (unborn) child’s health—may also worry about parenting when the child is born. They may feel less competent as a parent, or restricted in their activities because of their caretaking responsibilities, they may be more negative about their health, their relationship with their spouse, and their relationship with their child. These are all separate components of parenting stress (Abidin 1995). A general tendency during pregnancy to worry about pregnancy-related issues may continue after birth and transfer to negative perceptions of parenting-related issues, particularly those aspects that appear beyond one’s control. In a similar fashion, a high level of general anxiety as measured through trait or state anxiety symptoms during pregnancy may induce or carry over to parenting stress as well. In addition, symptoms of depression in the prenatal period could continue after birth and may also result in high levels of this aspect of parenting stress. Alternatively, children who were prenatally exposed to high levels of maternal anxiety or depression may express more difficult temperament from birth onwards, as some previous studies suggest (Huizink et al. 2002; Nolvi et al. 2016). This may increase levels of parenting stress in new mothers as well.

We therefore investigated the extent to which prenatal maternal mood factors, in particular symptoms of general anxiety, pregnancy-specific anxiety, and depression, were predictors of parenting stress at 3 months postpartum in a longitudinal study among 1073 first-time pregnant women. We explored whether both anxiety and depression symptoms during pregnancy were uniquely related to multiple aspects of parenting stress and therefore, we modeled the prospective associations between maternal pregnancy mood and all seven subscales of our parenting stress instrument. General anxiety, pregnancy anxiety, and depression are often intercorrelated, but may still have separate or independent impact on parenting stress. We therefore explored the independent prospective associations between levels of general anxiety, pregnancy-specific anxiety, and depression across pregnancy, with parenting stress 3 months after birth. Finally, we explored whether increase in mood scores from first to third trimester of pregnancy predicted parenting stress.

## Methods

### Participants

This study is part of the longitudinal cohort Generations^2^, which follows first-time pregnant mothers during pregnancy and after they give birth to their child to study the transition to parenthood. Participants were recruited through midwifery practices in the area of Amsterdam, the Netherlands. All women who visited cooperating midwifery practices for the first time received a recruitment letter and informed consent form. Women were eligible for inclusion when they were pregnant with their first child, did not receive a prenatal diagnosis for a congenital abnormality of the fetus after ultrasounds at 12 and 20 weeks gestation and were fluent in Dutch. When they filled out the questionnaires at the first assessments, they were approximately 12 weeks pregnant (target sample, *N* = 1355). For this study, we used the data from women who filled out questionnaires at three time points during pregnancy (at approximately 12, 22, and 32 weeks pregnant), and at one time point after giving birth (±3 months after birth). Written informed consent was obtained from all participants. These women were on average 29.6 years old (SD = 4.18) at first assessment. Sixty-five percent of the women had a higher educational level (higher professional education or university level), which is higher than women in the general Dutch population between age 25–35 (42–44% had a higher education in 2009–2011; (Statistics Netherlands 2015)). Most women had a partner (98.1%). The large majority (86.8%) had a Dutch background (i.e., both their parents and the women themselves were born in the Netherlands). Whenever a woman and/or her parents were not born in the Netherlands, the majority (±60%) was born in a Western country (e.g., Europe or North America; Statistics Netherlands 2015). The large majority of women (96.5%) reported that their baby was born healthy.

Analyses were performed on a sample of 1073 women (79% percent of the target sample) for whom pregnancy-specific anxiety scores at the third time point (t3, *M* = 32.4 weeks of pregnancy, SD = 3.89) and at least one of the other two earlier time points during pregnancy (i.e., t1, *M* = 13.7 weeks of pregnancy, SD = 3.17; t2, *M* = 22.2 weeks of pregnancy, SD = 2.77), parenting stress scores at t4 after child birth (t4, *M* = 14.0 weeks after child birth, SD = 3.19), as well as the control variables included in this study (described later in this section) were available.

### Measures

Parenting stress constructs were assessed at 3 months after birth (t4) with the Nijmeegse Ouderlijke Stress Index (NOSI; de Brock et al. 1992), the Dutch version of the Parenting Stress Index (PSI; Abidin 1995). The PSI assesses perceived parenting stress due to different sources, which can be parent- or child-specific domains. Only the parenting domain of the PSI—containing 58 self-report items—was assessed in this study because the child-specific domains could not be applied 3 months after birth. Mothers could respond on a 6-point Likert scale with scores ranging from 0 (totally disagree) to 5 (totally agree). The parenting domain consists of seven subscales: sense of competence (13 items; Cronbach’s alpha = .84; item example, I cannot take decisions without help). A high score on this scale reflects a poor sense of competence; role restriction (seven items; alpha = .83; example, I feel restricted because of my duties as a parent; attachment (seven items; alpha = .60; example, My child and I always have a good relationship, reverse-coded item). As with sense of competence, a high score on this scale also reflects poor attachment of the parent to the child; depression (12 items; alpha = .81; example, There are quite some things in my life that bother me); experience of health (six items; alpha = .75; example, Lately, I feel fine physically, reverse-coded item), with high scores reflecting poor experienced health; social isolation (six items; alpha = .69; example, I feel alone and without friends); and relationship with spouse (seven items; alpha = .74; example, Since the birth of this child, my partner gives me less support and help than I expected), with high scores reflecting poor relationship with spouse. Before calculating mean scores of the subscales, all items (six items) where a high score represented low parenting stress were reversed, so that high scores indicated high parenting stress. For each subscale, mean scores were then calculated by dividing the sum score across items with valid data by the number of valid items in the subscale. These mean scores ranged from 0 (low parenting stress) to 5 (high parenting stress) Note that the relationship with spouse subscale was not available for single mothers without a partner (1.5% of the final sample).

Pregnancy-specific anxiety was assessed at approximately 12 (t1), 22 (t2), and 32 (t3) weeks gestation with the Dutch version of the self-report Pregnancy-Related Anxieties Questionnaire-Revised (PRAQ-R; Huizink 2000; Huizink et al. 2004). The PRAQ-R is a shortened version of the PRAQ (Van den Bergh 1990), containing the 34 items of the original PRAQ with the highest factor loadings on each of the five subscales (i.e., fear of giving birth, fear of bearing a physically or mentally handicapped child, fear of changes and disillusion in partner relationship, fear of changes, and concern about one’s mental well-being and mother child-relationship). The PRAQ-R contains items such as “I am afraid of pain during the contractions and the child-bearing,” “I have thoughts our child will be infirm or weak,” “I am afraid my partner is unfaithful to me,” “I worry about my unattractive appearance,” and “I worry about the sudden changes of my mood.” Scores on each item ranged from 0 (absolutely not applicable) to 4 (very well applicable). Cronbach’s alpha for the total PRAQ scale ranged from .89 to .90 across the three time points. Total mean item scores for pregnancy-specific anxiety were computed, with a minimum of 0 (not anxious at all) and a maximum of 4.

State anxiety was measured with the Dutch adaptation of the State-Trait Anxiety Inventory (STAI; Spielberger et al. 1970) at t1-t3, which has been shown to be a reliable and valid measure in general populations but also in pregnancy (Gunning et al. 2010; Meades and Ayers 2011). The state anxiety scale of the STAI contains 20 items that ask the participant to describe how she feels at the moment, conceptualized as a transitory emotional state (in contrast to trait-anxiety, asking how she generally feels). Items in this scale were for example “I am tense,” “I am confused,” “I am worried,” “I feel calm,” and “I feel self-confident.” Women could respond on a 4-point Likert scale ranging from 1 (not at all) to 4 (very much). Some items were stated as an opposite of anxiety and scores were reversed, so that higher scores represented more anxiety. Cronbach’s alpha for the scale ranged from .93 to .94 across the three time points. Mean item scores with a minimum score of 0 (not anxious at all) and a maximum of 3 were calculated for the main analyses.

Trait anxiety was also measured with the STAI (Spielberger et al. 1970). The trait anxiety scale of the STAI contains 20 items that ask the participant to describe how she feels generally, referring to a relatively stable proneness to anxiety. Items in this scale were for example “I feel exhausted,” “I lack self-confidence,” “I feel nervous and restless,” “I feel calm and cool,” and “I feel at ease.” Women could respond on a 4-point Likert scale ranging from 1 (not at all) to 4 (very much). Like state anxiety, some item scores needed to be reversed, so that higher scores represented more anxiety. Cronbach’s alpha for the scale ranged from .92 to .93 across the three time points. Mean item scores with a minimum score of 0 (not anxious at all) and a maximum of 3 were calculated for the main analyses.

Depression was assessed with the Dutch version of the Beck Depression Index (BDI; Beck et al. 1961 *)* at t1-t3. The BDI consists of 21 items that assess the intensity of (symptoms of) depression, including “Sadness,” “Guilty feelings,” “Loss of energy,” “Irritability,” and “Indecisiveness.” Women could select one of out of four options, ranging from 0 (representing low intensity of depression; e.g., I do not feel sad, or I make decisions about as well as ever) to 3 (representing high intensity; e.g., I feel so sad or unhappy I can’t stand it, or I have trouble making any decisions). The BDI has also been validated for use in pregnancy (Holcomb et al. 1996). Cronbach’s alpha ranged from .82 to .84 across the three time points. For the analyses, the mean was computed of the 21 depression items, ranging from 0 (low intensity of depression) to 3.

Higher educational level, ethnicity, and having a partner were dummy-coded as, respectively, 1 = higher professional education or university degree, 0 = all other educational levels; 1 = both parents and participant born in the Netherlands, 0 = at least one parent and/or participant born elsewhere; and 1 = having a partner, 0 = no partner.

Life events were also assessed at 3 months after birth with the PSI (de Brock et al. 1992). The life events subscale consists of 40 items, in which mothers were asked about the presence of major/stressful life events (e.g., accident, marriage, death of a close family member, financial problems) in the last 12 months. When any of the life events occurred, the score was 1 for that life event, and 0 when the event did not occur. Because all women were pregnant and gave birth, these two items were excluded. The remaining 38 scores were summed.

Birth-related covariates were also assessed at 3 months after birth, including gestational age at birth, and interventions during labor (interventions, incision, surgical evacuation of placenta, artificial rupture of the membranes, labor induction in the hospital, artificial stimulation of contractions, suturing, vacuum extraction, cesarean section, planned caesarian section; labor interventions score = number of “yes” on these nine items).

### Statistical approach

Before performing the main analyses, non-response analyses, means, and standard deviations for the study variables at each time point were calculated, as well as correlations between the variables. The main analyses were performed in three steps. In the first step, latent scores were computed for the t1-t3 scores of each of the four mood variables separately (i.e., pregnancy-specific anxiety, state anxiety, trait anxiety, and depression), and tested in four separate models as predictors of the seven t4 parenting stress constructs. In the second step, the seven t4 parenting stress constructs were simultaneously regressed on the four latent t1-t3 mood scores. In the final step, difference scores between the observed t3 and the t1 mood scores were examined as predictors of the seven t4 parenting stress constructs. In each of these models, cross-sectional correlations were estimated between the t4 parenting stress constructs, and between the prenatal mood variables (in steps 2 and 3). To control for life events, duration of pregnancy, and interventions during labor, the t4 parenting stress constructs were also regressed on these variables. All parenting stress and mood variables were regressed on age, higher educational level, ethnicity, and having a partner.

All models were fitted in Mplus 7.3 (Muthén and Muthén 2010). Model fit was evaluated using the comparative fit index (CFI), the Tucker-Lewis index (TLI) with values >.90 indicating acceptable fit and values >.95 indicating close fit (Bentler and Bonett 1980), and the root mean square error of approximation (RMSEA) with values ≤.08 indicating acceptable fit and values ≤.05 indicating good fit (see Marsh et al. 2004). A robust maximum likelihood estimator (MLR), which produces robust standard errors, was used to account for the non-normal distribution of mood and parenting stress scores. Due to the large sample size and the number of tests performed in this study, alpha was set at .01.

## Results

### Non-response analysis

Seventy-nine percent of the target population was included in this study (1073 of 1355 women). Scores of the remaining 21% of the women were missing because they did not want to continue their participation during the follow-up or had missing scores at t3 and/or t4 without having quit. The included women significantly differed from the non-included women with respect to t1 pregnancy-specific anxiety (*F*(1, 1349) = 10.8, *p* < .01), t1 state anxiety (*F*(1, 1344) = 16.9, *p* < .001), t1 trait anxiety scores (*F*(1, 1345) = 14.8, *p* < .001), and t1 depression scores (*F*(1, 1345) = 6.82, *p* < .01). Non-included women had higher scores on each of the mood variables at t1 compared to women included in the 1073 sample. Moreover, included women were older on average than non-included women, *F*(1, 1342) = 20.8, *p* < .001. Women included in the 1073 sample more often had a higher educational level (χ^2^(1) = 36.6, *p* < .01) than non-included women. No other significant differences between included and non-included women were present.

### Descriptive statistics

The descriptive statistics in Table 1 show the mean item scores and standard deviations of pregnancy-specific anxiety (t1-t3), state anxiety (t1-t3), trait anxiety (t1-t3), depression (t1-t3), and the parenting stress constructs (t4). With respect to the covariates, the life events score was on average 2.08 (SD = 1.71). On average, babies were born 2 days prior to the due date (mean = −2.04, SD = 11.3). The median number of interventions during labor was 2.

**Table 1:** Descriptives of pregnancy-specific anxiety, state anxiety, trait anxiety, depression and the parenting stress constructs, and all the control variables

	*M*	SD
Pregnancy-specific anxiety (PRAQ total)		
t1 (≈12 weeks pregnant)	0.95	0.43
t2 (≈22 weeks pregnant)	0.94	0.44
t3 (≈32 weeks pregnant)	0.93	0.43
State anxiety		
t1 (≈12 weeks pregnant)	0.62	0.43
t2 (≈22 weeks pregnant)	0.55	0.44
t3 (≈32 weeks pregnant)	0.59	0.43
Trait anxiety		
t1 (≈12 weeks pregnant)	0.60	0.39
t2 (≈22 weeks pregnant)	0.54	0.41
t3 (≈32 weeks pregnant)	0.54	0.40
Depression		
t1 (≈12 weeks pregnant)	0.41	0.22
t2 (≈22 weeks pregnant)	0.37	0.21
t3 (≈32 weeks pregnant)	0.42	0.22
Parenting stress t4 (≈3 months after birth)		
Competence	0.67	0.56
Restriction	1.58	0.94
Attachment	0.42	0.44
Depression	0.74	0.62
Experience of health	1.18	0.89
Social isolation	0.90	0.72
Relation with spouse	1.17	0.79

Correlations between the study variables are shown in Table 2. Significant large positive correlations (Cohen 1988) between pregnancy-specific anxiety scores at the three time points were found. Moderate to large correlations were found for the state anxiety, trait anxiety, and depression scores at the three time points. In addition, correlations between the four different mood variables were significant at each time point, and across time points (ranging from *r* = .37 to *r* = .81). The seven parenting stress constructs at t4 also correlated significantly with each other (*r* range = .31 to .79), as well as the four t1-t3 mood variables and the t4 parenting stress variables (*r* range = .15 to .46).

**Table 2:** Correlations between pregnancy-specific anxiety, state anxiety, trait anxiety, depression, and parenting stress constructs across assessments

Variable		1.	2.	3.	4.	5.	6.	7.	8.	9.	10.	11.	12.	13.	14.	15.	16.	17.	18.
1.	PRAQ total t1	–																	
2.	PRAQ total t2	.75	–																
3.	PRAQ total t3	.74	.79	–															
4.	State anxiety t1	.56	.50	.49	–														
5.	State anxiety t2	.46	.57	.51	.60	–													
6.	State anxiety t3	.47	.52	.60	.61	.65	–												
7.	Trait anxiety t1	.56	.52	.51	.77	.57	.59	–											
8.	Trait anxiety t2	.51	.60	.55	.61	.79	.64	.74	–										
9.	Trait anxiety t3	.47	.54	.60	.59	.62	.81	.70	.78	–									
10.	Depression t1	.47	.43	.42	.58	.45	.44	.59	.52	.50	–								
11.	Depression t2	.43	.51	.47	.45	.61	.51	.49	.65	.58	.64	–							
12.	Depression t3	.37	.42	.50	.42	.47	.59	.45	.53	65	.61	.70	–						
13.	Competence t4	.36	.43	.41	.29	.33	.36	.35	.38	.38	.22	.26	.26	–					
14.	Role restriction t4	.30	.34	.34	.21	.21	.26	.26	.26	.30	.19	.19	.25	.58	–				
15.	Attachment t4	.28	.31	.30	.22	.25	.25	.29	.28	.26	.15	.17	.15	.63	.45	–			
16.	Depression t4	.41	.46	.45	.34	.35	.38	.44	.43	.45	.30	.32	.34	.79	.59	.60	–		
17.	Exp. of health t4	.31	.36	.36	.27	.31	.34	.32	.33	.36	.28	.30	.34	.49	.48	.36	.53	–	
18.	Social isolation t4	.36	.40	.40	.34	.32	.35	.38	.40	.41	.32	.36	.37	.51	.59	.40	.56	.48	–
19.	Relation spouse t4	.33	.36	.34	.29	.26	.27	.32	.33	.33	.25	.27	.26	.47	.54	.31	.47	.43	.57

Higher scores on the t4 parenting stress constructs indicate higher parenting stress. All entries are significant at *p* < .001

### Mood during pregnancy and parenting stress after giving birth

First, a model (model 1a) was fitted in which a latent score was estimated for pregnancy-specific anxiety across pregnancy (t1-t3). The seven t4 parenting stress constructs were regressed on the latent pregnancy-specific anxiety score, as well as life events and the birth-related control variables. This model had a good fit to the data (CFI = .99, TLI = .98, RMSEA = .03). Results in Table 3 show that pregnancy-specific anxiety positively predicted all parenting stress constructs after controlling for life events and birth-related factors. Subsequently, similar models were fitted with a latent score of state anxiety (model 1b), trait anxiety (model 1c), and depression (model 1d) respectively, as a predictor of the seven parenting stress constructs. All three models had a good fit (CFI ≥.99, TLI ≥.95, RMSEA ≤.04). Results of the three models in Table 3 show that latent t1-t3 state anxiety, trait anxiety, or depression scores were all significantly and positively predicting the seven parenting stress constructs. The *R* ^2^s shown in Table 3 indicate that the explained variance of the parenting stress constructs was generally higher in the models where the parenting stress constructs were regressed on anxiety as opposed to depression.

**Table 3:** Estimated pathways from latent t1-t3 scores of pregnancy-specific anxiety, state anxiety, trait anxiety, and depression to the t4 parenting stress constructs, and the explained variance (*R* ^2^) of the parenting stress constructs (models 1a to 1d)

Mood variables, latent t1-t3 scores	Pregnancy-specific anxiety (model 1a)				State anxiety (model 1b)				Trait anxiety (model 1c)				Depression (model 1d)			
	*B*	SE	*β*	*R* ^2^	*B*	SE	*β*	*R* ^2^	*B*	SE	*β*	*R* ^2^	*B*	SE	*β*	*R* ^2^
Parenting stress t4																
Competence	0.70	0.05	0.45	0.22	0.73	0.07	0.42	0.19	0.76	0.06	0.44	0.20	1.05	0.14	0.30	0.11
Role restriction	0.97	0.08	0.37	0.18	0.87	0.10	0.30	0.13	0.96	0.10	0.33	0.15	1.60	0.23	0.27	0.12
Attachment	0.42	0.04	0.34	0.12	0.43	0.07	0.31	0.11	0.45	0.05	0.33	0.12	0.53	0.10	0.19	0.05
Depression	0.85	0.06	0.49	0.26	0.86	0.07	0.45	0.22	0.97	0.07	0.51	0.27	1.46	0.15	0.38	0.17
Experience of health	0.92	0.08	0.37	0.18	1.01	0.10	0.37	0.17	1.01	0.10	0.37	0.17	1.93	0.20	0.35	0.15
Social isolation	0.85	0.06	0.43	0.21	0.93	0.09	0.41	0.20	1.01	0.08	0.46	0.23	1.91	0.18	0.43	0.21
Relation with spouse	0.82	0.07	0.37	0.17	0.80	0.09	0.32	0.14	0.89	0.08	0.37	0.16	1.46	0.18	0.30	0.12

Higher scores on the parenting stress constructs indicate higher parenting stress. All path coefficients were significant at *p* < .001

**Fig. 1:**
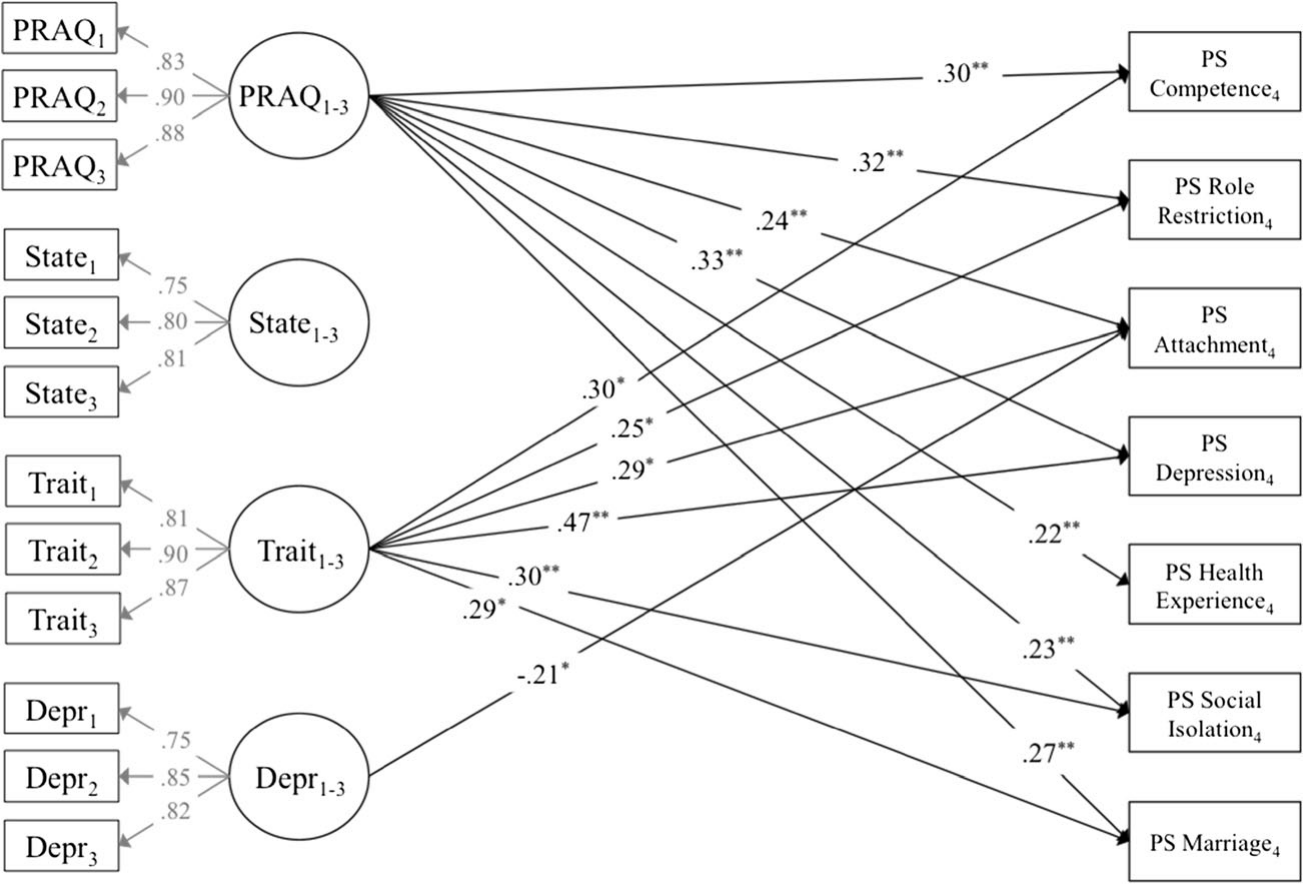
Standardized path estimates for the paths between the pre-birth mood variables and the post-birth parenting stress (PS) constructs in the model with t1-t3 latent pre-birth pregnancy specific anxiety (PRAQ), state anxiety (State), trait anxiety (Trait), and depression (Depr). Only significant (*p* < .01) links are shown. Correlational links are estimated between the latent t1-t3 mood variables, and between the seven t4 PS constructs. Residual variances are estimated for all variables. Higher scores on the parenting stress constructs indicate higher parenting stress. *p < .01, **p < .001

In the second step, the four separate models for the mood variables (models 1a–d) were combined in one model. The seven parenting stress constructs were regressed on the four latent t1-t3 mood variables. This model (model 2) had an acceptable fit to the data (CFI = .95, TLI = .91, RMSEA = .06). Results are shown in Fig. 1. Pregnancy-specific anxiety t1-t3 significantly and positively predicted all seven parenting stress constructs. Trait anxiety t1-t3 predicted the parenting stress constructs, except for health experience. In the combined model, links from state anxiety t1-t3 to the parenting stress constructs were no longer significant. Depression t1-t3 was only found to negatively predict the attachment parenting stress construct when tested simultaneously with the other mood variables (and allowing for correlational links between the latent mood variables). Results were largely similar with respect to significance and the direction of significant paths when t4 parenting stress constructs were regressed on the observed t1 mood variables or on the observed t3 mood variables instead of the latent t1-t3 mood constructs, indicating that—overall—time of assessment of mood during pregnancy hardly influenced the effects of prenatal mood on parenting stress.

In the final step, we explored whether change in mood scores during pregnancy had an effect on t4 parenting stress. The results from the model with t3 – t1 difference scores (model 3; CFI = 1.00, TLI = 1.00, RMSEA = .00, but note that the test of absolute model fit is also non-significant, *χ* ^*2*^(12) = 10.2, *p* = .60) showed that none of the paths from the t3 – t1 difference scores to the t4 parenting stress constructs reached significance (all *p*’s >.01). This indicates that changes in mood during pregnancy did not significantly affect t4 parenting stress.

## Discussion

This follow-up study from pregnancy onwards showed that higher levels of parenting stress were especially predicted by pregnancy-specific anxiety (all parenting stress subscales) and trait anxiety during pregnancy (six out of seven parenting stress scales). In contrast, neither prenatal depression nor state anxiety remained significant predictors of higher levels of parenting stress when tested simultaneously with the other prenatal mood variables, as opposed to when examined separately. These results were already alluded to in the first step of our analyses, showing that in separate models, pregnancy anxiety and trait anxiety explained more variance than did state anxiety and depression. This is an important finding, as it shows how maternal mood during pregnancy is related to the experiences and perceptions of mothers as a new parent as measured with the parenting stress scale. Furthermore, our study shows that particularly prenatal maternal anxiety—both general (trait) anxiety and pregnancy-specific anxiety—were important predictors of (almost) all aspects of parenting stress. It is therefore likely that women with a worried mind during pregnancy continue to worry as a parent of their newborn child as well and are thus susceptible to parenting stress. If women experience difficulty to cope with pregnancy and its related stressors, it can be expected that parenting is another major life transition that may be perceived as difficult to cope with, leading to a perceived discrepancy between the demands of parenting and the available resources to meet with those demands (Abidin 1995). Parenting stress may be the result, as our findings suggest.

It is of interest that we found that particularly maternal anxiety during pregnancy was uniquely related to parenting stress, taking into account maternal depression. In contrast, prenatal symptoms of maternal depression were not positively predictive of the parenting stress index subscales when anxiety scores were taken into account. Thus, examining anxiety and depression simultaneously showed us the relatively stronger and more consistent impact of both pregnancy-specific anxiety and trait anxiety on parenting stress in comparison to depression. This implies that it is important to take notice of maternal anxiety during pregnancy and include measures of both pregnancy-specific anxiety and trait anxiety when testing for (independent) effects of prenatal depression on maternal or parenting outcomes in further studies. A previous study of our group showed no differential association between prenatal maternal depression or prenatal maternal general anxiety with (postpartum) parenting self-efficacy, which reflects the expectations of the mothers about their ability to parent successfully (Kunseler et al. 2014). Another study has related both depression and anxiety levels during pregnancy with higher overall levels of parenting stress (Misri et al. 2010), but both this study and that of Kunseler et al. (2014) did not include pregnancy-specific anxiety measures. It has been suggested that this particular measure of anxiety during pregnancy is more sensitive to capture to mood variability than less context-dependent questions (Robertson Blackmore et al. 2016), such as the depression items of the BDI or questions on general anxiety. This may partly explain why pregnancy-specific anxiety better predicts parenting stress than prenatal maternal depression in our study and shows independent contributions to parenting stress scores when trait anxiety is taking into account as well. Indeed, pregnancy-specific anxiety seems to be a robust and independent predictor of birth outcome, but also of maternal mood in the postpartum period. Robertson Blackmore et al. (2016) suggested that anxieties specifically related to pregnancy can be regarded as distinct clinical phenotype, predicting postnatal mood disturbance. Our study findings add to this evidence by showing that pregnancy-specific anxiety also has independent associations with parenting stress, when controlling for both general anxiety measures and depression.

The depression subscale of the PSI was not related to prenatal depression scores when anxiety scores were taken into account, but a close examination of the items may explain why: several items of the parenting stress subscale on depression focus on feelings of guilt or a lack of confidence, which may not be specific for feelings of depression, but could also be linked to anxiety. Correlation coefficients in Table 2 further support this, as all prenatal pregnancy-specific and trait anxiety measures were stronger correlated (*r*’s ranging between 0.41–0.46) to the subscale depression of the PSI than BDI (*r*’s = 0.30–0.34) was. Thus, the items of the prenatal depression scale of the well-validated BDI are not easily comparable with the depression subscale of the PSI after birth, and therefore, the PSI may have captured a different and more general construct of negative thinking, rather than core depression.

Nonetheless, it is striking that compared to prenatal depression, two aspects of anxiety—pregnancy-specific anxiety and trait anxiety—were independent and better predictors of (almost) all subscales of parenting stress, when each of the maternal mood factors were modeled simultaneously. It is of interest that our results imply the importance of including measures of pregnancy-specific anxiety alongside general trait anxiety, when predicting postnatal (maternal) outcomes, as its predictive validity is further supported by our study. It may be that the instrument on pregnancy-specific anxiety is particularly sensitive in capturing individual vulnerability to stressors and anxiety related to specific transitional demands that arise during pregnancy and therefore is related to a variety of postnatal birth related, maternal and child outcomes (Glover 2014), including our measure of parenting stress.

Of course, our results are based on a general population of pregnant women who were relatively highly educated, and who had a relatively low to normal risk for clinical levels of depression and anxiety. Given the results of the non-response analysis, we could have underestimated the strengths of the associations between prenatal maternal mood and parenting stress to some extent. Therefore, the findings of this study may not apply to women with more severe levels of depression and anxiety during pregnancy or for women from lower SES strata. Besides this limitation of our study, several other aspects of the current study should be carefully considered. First, despite the longitudinal design of our study, the reported associations do not reflect causality. For instance, a general tendency to worry may be expressed as pregnancy anxiety first, and as parenting stress later. It is also possible that newborn babies show more difficult temperament, more crying behavior, and a less predictable sleeping schedule after being exposed to high levels of prenatal maternal anxiety or depression (e.g., Van den Bergh et al. 2005; Huizink et al. 2002). This infant behavior may lead to parenting stress (Bates 1980; Chang et al. 2004), and hence, the findings of this study could reflect indirect pathways rather than direct ones. We did, however, control for the experience of life events, and some birth-related variables, gestational age at birth and interventions during labor, to adjust for the effects that these factors may have had on parenting stress. Second, we did not focus particularly on potential timing effects of prenatal maternal mood on parenting stress, but rather focused on a more global prediction of prenatal maternal mood across pregnancy and changes in maternal mood in relation to parenting stress. Future studies may specifically examine whether particular high levels of maternal anxiety or depression during specific periods of pregnancy may be predictive of high parenting stress after birth. We did, however, test whether changes in prenatal mood between the first and last trimester affected parenting stress 3 months postpartum. This did not yield any significant associations between the difference scores and the parenting stress measures. Third, the parenting stress subscale attachment has a relatively low internal consistency, and findings related to this scale should be interpreted with caution.

Strengths of this study include the large study sample, and the repeated assessments of prenatal maternal mood factors, and the latent simultaneously modeling approach to test for the independent predictive effect of anxiety and depression.

To conclude, the current study showed that particularly pregnancy-specific anxiety and trait anxiety predict higher levels of parenting stress 3 months after birth. It is important to better understand how the combined effects of prenatal exposure to maternal anxiety and postnatal exposure to parenting stress may further impact the lives of young parents and their children. This study shows that already during pregnancy, we can identify women with high levels of particularly trait and pregnancy-specific anxiety, which could continue in or foreshadow higher levels of parenting stress. Several recent studies tested intervention programs targeting maternal anxiety in pregnancy, for instance using mindfulness (Matvienko-Sikar et al. 2016), or music stimulation (García González et al. 2017), with promising results. These early prevention and intervention to reduce maternal anxiety in pregnancy could hold the key for a more advantageous trajectory of early postnatal parenting and, indirectly, for a more optimal environment of the developing child.
